# In this month’s Bulletin

**DOI:** 10.2471/BLT.24.001124

**Published:** 2024-11-01

In the editorial section, Shyama Kuruvilla et al. (770) call for papers for a theme issue on traditional medicine and global health. Mekdes D Feyssa (771) describes the intersection of WHO’s normative mandate with the evolution of Ethiopia’s health sector.

Gary Humphreys (774–775) reports on how declining total fertility rates in urban areas are causing a re-examination of pro-natal policies in several countries. Hamid Jafari talks to Gary Humphreys (776–777) about the non-linear nature of progress towards polio eradication and the challenges faced in the last two polio-endemic countries in the world.

## Brazil

### Single cigarette sales 

André Salem Szklo et al. (834–836) find contraventions of tobacco control policies.

## China 

### Evaluating a road safety campaign

Peishan Ning et al. (786–794) observe behaviours of motorcyclists and bicyclists

## Philippines 

### Documenting unmet need for contraception

Miguel Antonio Garcia Estrada et al. (778–785) collect legal evidence.

## Portugal 

### Political parties' health proposals 

Sara Machado et al. (820–827) propose an approach to health system assessment.

## WHO European Region

### Football matches in a pandemic

Tanja Schmidt et al. (803–812) examine lessons learned during the COVID-19 response.

## Global 

### Getting insulin products to market

Henry MJ Leng and Jicui Dong (795–802) detail barriers to WHO prequalification.

### Dealing with a food crisis

Anne Marie Thompson Thow (813–819) offers policy considerations for preventing malnutrition.

### Training videos for respiratory equipment 

Joshua Tim et al. (828–833) show how participants from 189 countries contributed to content development.

### Geographical access to emergency obstetric services 

Aduragbemi Banke-Thomas et al. (837–839) argue for a rethink on proximity as a measure of access in urban areas.

